# Cross potential selection: a proposal for optimizing crossing combinations in recurrent selection using the usefulness criterion of future inbred lines

**DOI:** 10.1093/g3journal/jkae224

**Published:** 2024-09-23

**Authors:** Kengo Sakurai, Kosuke Hamazaki, Minoru Inamori, Akito Kaga, Hiroyoshi Iwata

**Affiliations:** Graduate School of Agricultural and Life Sciences, University of Tokyo, Tokyo 113-8657, Japan; Molecular Informatics Team, RIKEN Center for Advanced Intelligence Project (AIP), RIKEN, Chiba 277-0871, Japan; Graduate School of Agricultural and Life Sciences, University of Tokyo, Tokyo 113-8657, Japan; Soybean and Field Crop Applied Genomics Research Unit, Institute of Crop Science, National Agriculture and Food Research Organization, Tsukuba 305-8518, Japan; Graduate School of Agricultural and Life Sciences, University of Tokyo, Tokyo 113-8657, Japan

**Keywords:** cross selection, short-term genetic improvement, breeding strategy, progeny distribution

## Abstract

In plant breeding programs, rapid production of novel varieties is highly desirable. Genomic selection allows the selection of superior individuals based on genomic estimated breeding values. However, it is worth noting that superior individuals may not always be superior parents. The choice of the crossing pair significantly influences the genotypic value of the resulting progeny. This study has introduced a new crossing strategy, termed cross potential selection, designed to expedite the production of novel varieties of inbred crops. Cross potential selection integrates fast recurrent selection and usefulness criterion to generate novel varieties. It considers the segregation of each crossing pair and computes the expected genotypic values of the top-performing individuals, assuming that the progeny distribution of genotypic values follows a normal distribution. It does not consider genetic diversity and focuses only on producing a novel variety as soon as possible. We simulated a 30-year breeding program in 2 scenarios, low heritability ($h^{2}=0.3$) and high heritability ($h^{2}=0.6$), to compare cross potential selection with 2 other selection strategies. Cross potential selection consistently demonstrated the highest genetic gains among the 3 strategies in early cycles. In the 3rd year of the breeding program with a high heritability ($h^{2}=0.6$), cross potential selection exhibited the highest genetic gains, 138 times that of 300 independent breeding simulations. Regarding long-term improvement, the other selection strategies outperformed cross potential selection. Nevertheless, compared with the other 2 strategies, cross potential selection achieved significant short-term genetic improvements. Cross potential selection is a suitable breeding strategy for the rapid production of varieties within limited time and cost.

## Introduction

Plant breeding aims to enhance the genotypic value of a target trait through selection and crossing. The process of selecting candidates for plant breeding is important because it directly influences the outcomes of breeding programs. Genomic prediction (GP) models have been developed to aid in selecting superior candidates using genome-wide polymorphism data (Meuwissen *et al*. 2001). These models were constructed using training data, comprising genome-wide marker and phenotypic data, to estimate the effects of markers across the genome on a target trait. By leveraging GP models, the genomic estimated breeding values (GEBVs) can be obtained for novel genotypes without the need to conduct field trials. This approach, known as genomic selection (GS), enables the selection of superior candidates based on the GEBVs.

In plant breeding, once candidates are selected, the next step involves determining the crossing pairs to generate progeny. As crossing pairs directly influence the genotypic values of the progeny, various crossing strategies have been devised to select crossing pairs from the current population. Genetic diversity plays a crucial role in driving genetic gains in breeding programs (Sanchez *et al*. 2023, 2024). We need to balance genetic gain and variance to achieve long-term genetic improvements. Thus, some selection methods balance the expected genotypic values of the next generation with a degree of kinship among the selected individuals (Wray and Goddard 1994; Meuwissen 1997). In addition, optimal cross selection (OCS) can balance genetic gain and genetic variance as well as select practical crossing pairs when considering the restrictions on crossing, such as sex groups and the number of crossing pairs per candidate (Kinghorn 2011). In addition, the usefulness criterion (UC) was introduced as a selection index for crossing pairs (Schnell and Utz 1975). UC represents the expected value of the superior fraction when producing inbred progeny from each crossing pair. It is defined as UC=μ+ihσ, where *μ* is the mean genotypic values of the cross, *i* is the selection intensity, *h* is the square root of heritability, and *σ* is the square root of genetic variance for the inbred progeny. *σ* of each cross was estimated by simulated inbred progeny using the estimated marker effect and genome-wide marker data of each cross (Lian *et al*. 2015; Mohammadi *et al*. 2015; Yao *et al*. 2018). In addition, in a 2-way cross, the genetic variance of inbred progeny can be computed using the recombination rates between 2 markers, estimated marker effects for the target trait, and the marker data of each cross (Lehermeier *et al*. 2017). Allier *et al*. (2019a) extended this formula to 3- and 4-way crosses. In breeding simulations, genetic improvements in the F_5_ population produced from crosses selected using UC were greater than those produced from crosses selected using GEBVs (des Déserts *et al*. 2023). In real fields, the accuracy of the estimated variance of inbred progeny is very low in maize (Lian *et al*. 2015; Adeyemo and Bernardo 2019), but in barely, large sample size enables the estimation of the variance of inbred progeny (Neyhart and Smith 2019). Additionally, in 6-row spring barely, the usefulness of UC, calculated using the estimated variance of the inbred progeny, has been verified in the field (Mohammadi *et al*. 2015). Crossing selection based on UC has significant potential for enhancing genetic improvement in plant breeding programs.

Gaynor *et al*. (2017) introduced a 2-part strategy to enhance the efficiency of plant breeding programs. This strategy comprises a “product development component,” which identifies promising individuals for release as varieties, and a “population improvement component,” which boosts the genotypic values of the breeding population through rapid recurrent selection conducted twice a year. Gorjanc *et al*. (2018) applied OCS to the population improvement component in a 2-part strategy and demonstrated substantial long-term genetic improvements compared with the standard 2-part strategy that selects candidates based on GEBVs. However, only a few breeding strategies have surpassed GS in achieving higher short-term genetic improvements. Herein, short-term refers to 20 or fewer cycles of recurrent selection. In recurrent selection, several selection strategies that consider the allele states of parents have been developed, but they do not fix the alleles that are needed for inbred crops (Akdemir and Sánchez 2016; Moeinizade *et al*. 2019; Bijma *et al*. 2020). Allier *et al*. (2019b) employed UC in recurrent selection and achieved higher short-term genetic improvement than GS. However, their breeding scheme was limited to crop species capable of producing doubled haploids (DHs). Furthermore, rapid recurrent selection was not employed in their breeding scheme, as their focus was not on expediting variety development. In practical breeding scenarios, it is necessary to expedite production of novel varieties. Therefore, it is imperative to develop breeding strategies that can achieve considerable short-term genetic improvements to rapidly produce novel varieties. However, such breeding strategies are yet to be developed.

This study introduced a novel breeding strategy for the rapid production of novel varieties. We proposed a new strategy termed cross potential selection (CPS), which integrates the UC and a fast recurrent selection. To assess the efficacy of CPS, we conducted 300 independent breeding simulations based on a 2-part strategy, comparing the genetic gains of the 3 breeding strategies: GS, OCS, and CPS. Additionally, we analyzed the genetic variance and fixation of beneficial alleles within the population improvement component across strategies to delineate the unique characteristics of CPS. The usefulness of UC has been reported to be partly dependent on the accuracy of the GP model (Lehermeier *et al*. 2017). We simulated 2 scenarios in this study: low heritability ($h^{2}=0.3$) and high heritability ($h^{2}=0.6$), and evaluated 3 breeding strategies for each scenario.

## Materials and methods

### Population in breeding simulations

The population used in breeding simulations was generated using whole-genome sequence data from a diverse panel of 198 soybean accessions. These accessions primarily comprise the Japanese and global soybean mini-core collections (Kaga *et al*. 2012; Kajiya-Kanegae *et al*. 2021). The whole-genome sequence data encompassed 4,776,813 single-nucleotide polymorphisms (SNPs) distributed across 20 pairs of chromosomes. SNPs that were heterozygous or had >95% missing data were excluded, along with those with a minor allele frequency < 0.1. Additionally, to avoid the accumulation of many SNPs in a particular chromosomal region, SNPs were filtered based on linkage disequilibrium, with pairs < 0.6 selected, resulting in a final set of 61,426 SNP markers. From a pool of 61,426 SNPs, we randomly selected 4,000 SNPs (200 SNPs per chromosome) for each independent breeding simulation to conserve computer memory and accelerate the simulations. We assumed that each chromosome had a length of 1 Morgan and that there was a linear relationship between map distances and physical distances. Consequently, the linkage map positions were calculated based on the physical positions of adjacent SNPs.

In each independent breeding simulation, of the 4,000 SNPs, 1,000 were randomly designated as quantitative trait nucleotides (QTNs) and nonzero effects were assigned to the genotypes of the 198 soybean accessions. The effect of QTNs followed a multivariate normal distribution.


(1) β∼MVN(0,0.35I)

where β represents the vector of the effect for the 1,000 QTNs, and I denotes the identity matrix. The effects of the remaining 3,000 SNPs were set to zero. The simulation setup (simulation of the linkage map positions and a variance of QTN) was adopted from Diot and Iwata (2023).

Each breeding simulation commenced with 150 individuals, generated from a 4-way cross. In the 4-way cross, we can mix 4 types of alleles effectively using 2 types of F_1_ genotypes. In the initial population, both high genetic diversity and genetic ability are required. A diverse panel of 198 soybean accessions was divided into 4 groups based on 4,000 SNPs using the k-means clustering algorithm. In each cluster, the accession with the highest genotypic value was selected as a parent for a 4-way cross. The 4-way cross comprised the 4 selected accessions and yielded 150 individuals from hybridizations between 2 different F_1_ parents.

### Breeding program

**Fig. 1.**
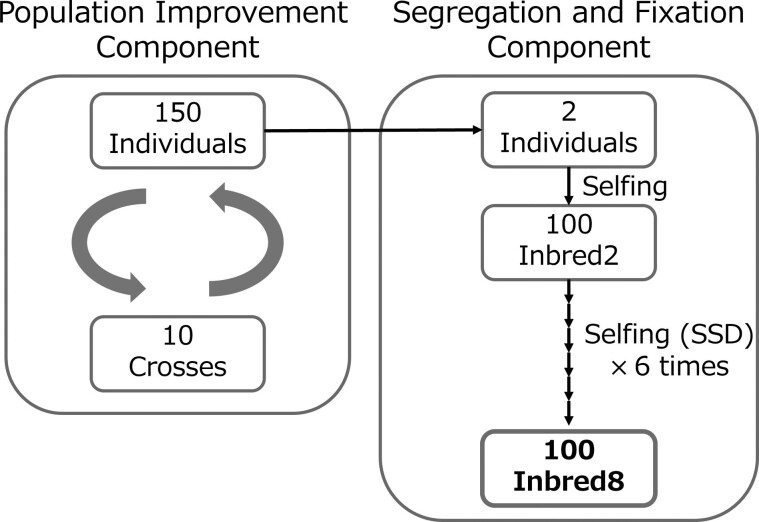
Overview of a breeding program adopted in this breeding simulation. SSD, single-seed decent.

In the breeding program, we adopted the 2-part strategy proposed by Gaynor *et al*. (2017) and adapted it for use in inbred crops that lack established protocols for producing DHs. Genotypes of inbred crops that cannot produce DH must be fixed using repeated selfing. As the number of selfings increased, the genetic variance of the recombinant inbred line (RIL) progeny increased. RIL progeny variance converges toward DH progeny variance after 5 rounds of selfing (Allier, *et al*. 2019a). The program comprises “population improvement component” and “segregation and fixation component,” (Fig. 1). The population improvement component aimed to enhance the genotypic value of a population through rapid recurrent selection. Mating and selection were performed by highly heterozygous individuals in the population improvement component. The segregation and fixation component involved repeated selfing to segregate and fix alleles. The primary objective of this breeding program was to develop genotypes with high genotypic values in the Inbred8 generation for subsequent release as varieties. In our study, the Inbred8 generation means the generation after 7 rounds of selfing.

For the population improvement component, each breeding strategy selected 10 crossing pairs from 150 individuals in each cycle (Fig. 1). Owing to limitations in the number of flowers and amount of pollen collected from each soybean plant, we restricted each individual to be used up to twice for crossing pairs in all cycles. In soybeans, as each individual has multiple flowers, each individual can become both a pollen and seed parent. Each crossing pair produced 15 progeny, resulting in 150 individuals per cycle. In the subsequent cycle, 10 crossing pairs were selected from a pool of 150 individuals, generating another 150 individuals. This iterative process of selection and crossing enhanced the genotypic potential of a population within the population improvement component.

For the segregation and fixation component, we selected 2 individuals annually from the 150 individuals in the population improvement component. The selected individuals underwent 7 rounds of selfing to segregate and fix the alleles, which fixed >99% of alleles. The first round of selfing yielded 50 progeny for each individual to facilitate allele segregation, resulting in 100 Inbred2 individuals. Subsequent selfing rounds were conducted using single-seed descent (SSD) method to fix the alleles. Ultimately, 100 Inbred8 individuals were produced annually, and their true genotypic values were used for strategy evaluation.

The efficacy of breeding programs depends on the selection and crossing strategies employed in population improvement components. To evaluate the performance of the program, we simulated a 30-year breeding program and compared 3 breeding strategies (GS, OCS, and CPS). Since the days to maturity of ‘Enrei’, which is one of the most famous Japanese soybean cultivars, ranges from 102 to 132 d (Yamada *et al*. 2012), about 2 cycles correspond to a year using a growth chamber. In the population improvement component, selection and crossing cycles were conducted twice a year. Throughout the 30-year breeding program, 60 selection and crossing cycles were carried out, resulting in 100 Inbred8 individuals on 30 occasions. The time required to produce 100 Inbred8 individuals was not considered in this simulation. In practical breeding, the utilization of generation advancement techniques enables a reduction in this timeframe.

### Simulation of phenotypic values and estimation of marker effects

We simulated phenotypic values of the initial 150 individuals for each simulation.


(2) $$\begin{array}{c} y_{i}=u_{i}+\epsilon_{i}\; \end{array}$$

where $y_{i}$ represents the phenotypic value of individual i(i=1,…,N), *N* is the number of individuals in the initial population (N=150), $u_{i}$ represents the true genotypic value, and $\epsilon_{i}$ represents the residual value. $u_{i}$ is computed as $u_{i}=\sum_{l=1}^{L}\;x_{i\;l}\beta_{l}$, where $x_{i\;l}$ denotes the SNP marker score of individual *i* on SNP marker *l*, encoded with −1, 0, 1 for the reference SNP marker, $\beta_{l}$ represents the true marker effect on SNP marker *l*, and *L* is the total number of SNP markers (L=4,000). $\varepsilon =(\epsilon_{1},\ldots ,\epsilon_{N}{)}^{T}$ follows the multivariate normal (MVN) distribution $\mathrm{MVN}(0,\;I\sigma_{\epsilon }^{2})$, where I is the N×N identical matrix and $\sigma_{\epsilon }^{2}$ is the residual variance. $\sigma_{\epsilon }^{2}$ is computed as $\sigma_{\epsilon }^{2}=\frac{\sigma_{g}^{2}}{h^{2}}-\sigma_{g}^{2}$, where $\sigma_{g}^{2}$ is the genetic variance and $h^{2}$ is the heritability in each scenario $\left(h^{2}=0.3\;\mathrm{or}\;0.6\right)$. $\sigma_{g}^{2}$ is computed as $\sigma_{g}^{2}=\frac{1}{N}\sum_{i=1}^{N}\;(u_{i}-\bar{u}{)}^{2}$, where $\bar{u}$ is mean genotypic value and computed as $\bar{u}=\frac{1}{N}\sum_{i=1}^{N}\;u_{i}$. By following these steps, phenotypic values can be simulated according to arbitrary heritability.

We built a G-BLUP model using phenotypic values and 4,000 SNP marker scores from the initial 150 individuals to estimate GEBVs. G-BLUP model was built using the “EMM.cpp” function in the “RAINBOWR” package in R v0.1.29 (Hamazaki and Iwata 2020). Marker effects were computed using the GEBVs (Wang *et al*. 2012) as follows:


(3) $$\begin{array}{c} \hat{\beta }={X_{\mathrm{init}}}^{T}{\left(X_{\mathrm{init}}{X_{\mathrm{init}}}^{T}\right)}^{-1}{\hat{u}}_{\mathrm{init}} \end{array}$$

where $\hat{\beta }$ represents the L×1 length estimated marker effects vector, $X_{\mathrm{init}}$ represents the N×L SNP marker score matrix of the initial population, and ${\hat{u}}_{\mathrm{init}}$ represents the N×1 length GEBV vector of the initial population. Marker effects were estimated once using the initial 150 individuals in each independent simulation.

### GS

This strategy involved selecting 10 individuals with the highest GEBVs, which were calculated using the following formula.


(4) $$\begin{array}{c} g_{i}=\overset{L}{\underset{l=1}{\sum }}\;x_{i\;l}\hat{\beta_{l}} \end{array}$$

where $g_{i}$ represents GEBV of individual i(i=1,…,N), *N* is the number of individuals (N=150), $x_{i\;l}$ denotes the SNP marker score of individual *i* on SNP marker *l*, $\hat{\beta_{l}}$ represents the estimated SNP marker effect on SNP marker *l*, and *L* is the total number of SNP markers (L=4,000). This strategy selected 10 individuals with the highest GEBVs and randomly determined 10 crossing pairs. Each individual was used twice and duplicate crosses were not permitted.

### OCS

This method selects 10 crossing pairs directly from the current population, considering the GEBVs and genetic diversity of the selected individuals (Gorjanc *et al*. 2018; Allier, *et al*. 2019b). This method involves solving the following optimization problem.


(5) $$\begin{array}{c} \max\overset{\mathrm{nc}}{\underset{k=1}{\sum }}\;a_{k}\mu_{k} \end{array}$$

(6) $$\begin{array}{c} \mathrm{with}\;D_{\mathrm{sel}}>\mathrm{He}(t) \end{array}$$

where nc is the number of total possible crosses $\left(\mathrm{nc}=\frac{N(N-1)}{2}\right)$, $a_{k}$ is a dummy variable for each cross (where $a_{k}=0$ indicates that cross *k* is not chosen and $a_{k}=1$ indicates that cross *k* is chosen), $\mu_{k}$ is the mean GEBV of cross *k*, which can be computed as $\mu_{k}=\frac{z_{k}^{T}g}{2}$, where g is the N×1 length GEBV vector calculated in Eq. 4, and $z_{k}$ is the N×1 length vector linking the cross *k* to the selected 2 individuals. $D_{\mathrm{sel}}$ is the genetic diversity of the selected crossing pairs, He(t) is the genetic diversity constraint at cycles t(t=1,…,T), and *T* is the final cycle of the population improvement component (T=60). $D_{\mathrm{sel}}$ and He(t) are defined as follows:


(7) $$\begin{array}{c} D_{\mathrm{sel}}=1-c^{T}Kc \end{array}$$

(8) $$\begin{array}{c} \mathrm{He}(t)=\{\begin{array}{c} He^{0}+{\left(\frac{t}{t^{⋆}}\right)}^{s}(He^{⋆}-He^{0}),\;\;t\leq t^{⋆}\\ He^{⋆},\;\;t>t^{⋆} \end{array} \end{array}$$

where c is the N×1 length individual contribution vector, computed as $c=\frac{1}{20}Za$ with $a=(a_{1},\ldots ,a_{\mathrm{nc}}{)}^{T}$ and $Z=(z_{1},\ldots ,z_{\mathrm{nc}})$, K is the N×N identical-by-state matrix, and $He^{0}$ is the genetic diversity of the initial population. Eq. 8 was originally put forward by Allier, *et al*. (2019b) and $t^{⋆}$, *s*, and $He^{⋆}$ are parameters in OCS. $t^{⋆}$ is the target cycle, *s* is the shape parameter, and $He^{⋆}$ is the remained genetic diversity in the target cycle. He(t) should reach $He^{⋆}$ when *t* is greater than or equal to $t^{⋆}$. *s* is a parameter that determines the trajectory of He(t). In this breeding simulation, we set $t^{⋆}=60$, s=1, and $He^{⋆}=\;0.01He^{0}$ for achieving long-term genetic improvement. K and $He^{0}$ were defined as follows:


(9) $$\begin{array}{c} K=\frac{1}{2}\left(\frac{1}{L}XX^{T}+1\right) \end{array}$$

(10) $$\begin{array}{c} He^{0}=\frac{1}{L}\overset{L}{\underset{\;j=1}{\sum }}\;2p_{j}^{0}(1-p_{j}^{0}) \end{array}$$

where *L* is the total number of SNP markers (L=4,000), X is the N×L SNP marker score matrix, and $p_{j}^{0}$ is the frequency of the referent allele in the initial population.

In each cycle, 10 crossing pairs were selected, and each individual could be used up to twice for crossing pairs. The restrictions are defined as follows:


(11) $$\begin{array}{c} \overset{\mathrm{nc}}{\underset{k=1}{\sum }}\;a_{k}=10 \end{array}$$

(12) $$\begin{array}{c} \max Za\leq 2 \end{array}.$$

In the OCS, we maximized Eq. 5 under Eqs. 6, 11, and 12. This optimization problem corresponds to a quadratic programming problem. We derived an approximate solution to this optimization problem using the heuristic algorithm developed by Sanchez *et al*. (2023). A heuristic algorithm does not guarantee optimality; however, it can obtain a solution that is close to the optimal solution at a certain level. In addition, GS and OCS only select individuals and do not consider crossing pairs because these strategies evaluate the genotypic ability of each individual. The best crossing pairs should be selected to increase the genetic ability of the next generation; however, our breeding program was designed to continuously increase the genetic ability. Therefore, GS and OCS randomly determined the crossing pairs in this study.

### CPS

CPS is a novel strategy that selects crossing pairs while considering the segregation of the target generation. It was important to improve the genetic abilities of 150 individuals in the population improvement component, and GS and OCS focused on genetic improvement in the population improvement component. However, the aim of this breeding program was to produce an Inbred8 individual with high genotypic value. Therefore, crossing pairs were selected based on the expected value of the superior fraction of the Inbred8 generation progeny. When we have phased SNP marker data for each individual, it is possible to estimate the genetic variance in the inbred progeny for any crosses by considering crosses between heterozygous individuals (not pure lines) as 4-way crosses of pure lines (Allier, *et al*. 2019a). Following the methodology outlined by Allier *et al*. (2019a), the genetic variance of the Inbred8 population for each cross was computed using the SNP marker effect and score.


(13) $$\begin{array}{c} \sigma_{k}^{2}={\hat{\beta }}^{T}\Sigma_{k}\hat{\beta } \end{array}$$

where $\hat{\beta }$ is the L×1 length estimated marker effect vector, and $\Sigma_{k}$ is the L×L variance-covariance matrix, computed from the SNP marker score of cross *k*. Each element of $\Sigma_{k}$ represents the variance or covariance between 2 markers at the Inbred8 generation. Further details on the calculation of this $\Sigma_{k}$ matrix for each cross are provided in File S1. In this breeding program, each selected cross produced 15 individuals, and the selected individuals for the population improvement component yielded 50 Inbred8 individuals. When selecting the best individual for the population improvement component from the 15 individuals, the expected maximum value of the Inbred8 individual for each cross was determined as follows:


(14) $$\begin{array}{c} UC_{k}=\mu_{k}+i_{\mathrm{cross}}h\sigma_{k} \end{array}$$

where $\mu_{k}$ is the mean GEBV of cross *k*, $i_{\mathrm{cross}}\approx 3.004$ is a selection intensity that corresponds to selecting the highest Inbred8 individual from the pool of 750 Inbred8 individuals (15 individuals × 50 Inbred8 individuals), and *h* is a square root of heritability. Following Zhong and Jannink (2007), we considered h=1 because we wanted the crosses to produce outstanding progenies. Using $UC_{k}$, we assessed the potential of each cross to produce outstanding individuals in the Inbred8 population. The objective of the CPS is defined as follows:


(15) $$\begin{array}{c} \max\overset{\mathrm{nc}}{\underset{k=1}{\sum }}\;a_{k}UC_{k} \end{array}.$$

In the CPS, we maximize Eq. 15 subject to the constraints outlined in Eqs. 11 and 12. Because this optimization problem corresponds to an integer programming problem, we can solve it and obtain the optimal solution. We solved this optimization problem using the “lp” function in the “lpSolve” package version 5.6.18 (Berkelaar *et al*. 2023 ) in R version 4.1.2.

### Selection for the segregation and fixation component

For the segregation and fixation component, we selected 2 individuals annually from the 150 individuals in the population improvement component. The same selection method was used in all 3 strategies. In this breeding program, selected each individual produced 50 Inbred8 individuals. We computed the UC for Inbred8 generation as follows:


(16) $$\begin{array}{c} \mathrm{UC}S_{i}=g_{i}+i_{\mathrm{self}}\sigma_{i} \end{array}$$

where $g_{i}$ is the same for Eq. 4, $i_{\mathrm{self}}\approx 2.054$ is a selection intensity that corresponds to selecting the highest Inbred8 individual from the pool of 50 Inbred8 individuals, and $\sigma_{i}$ is the genetic variance of the Inbred8 population for each individual *i*. Details on the calculation of this $\sigma_{i}$ for each individual are provided in File S1. We selected 2 individuals with the highest $\mathrm{UC}S_{i}$ values for the segregation and fixation component. Additionally, these 2 individuals can be used as crossing pairs in the population improvement component.

### Comparison

We conducted 300 independent breeding simulations for all 3 breeding strategies (GS, OCS, and CPS) for each scenario $\left(h^{2}=0.3\;\mathrm{or}\;0.6\right)$. In each independent breeding simulation, we selected 4,000 SNP markers and the loci of 1,000 QTNs, and simulated the effects of the QTNs. The primary objective of this breeding program was to generate individuals with high genotypic value in the Inbred8 generation for subsequent release. Following Allier *et al*. (2019b), we computed the genetic gains in the Inbred8 population as follows:


(17) $$\begin{array}{c} G^{I8}(t)=\frac{u^{I8}(t)-\;u^{I8}(t=0)}{\sqrt{\sigma_{g}^{2}}} \end{array}$$

(18) $$\begin{array}{c} u^{I8}(t)=\underset{i\in [1,N]}{\max}\;u_{i}^{I8}(t),\;\;N=\{\begin{array}{c} \;150,\;\;t=0\\ 100,\;\;2\leq t \end{array} \end{array}$$

where $u_{i}^{I8}(t)$ is the genotypic value of individual *i* in the Inbred8 population produced by the 2 selected individuals from the population improvement component at cycle t(t=2,4,…,60), $u^{I8}(t=0)$ is the maximum genotypic value of initial 150 individuals, *N* is the number of individuals, and $\sigma_{g}^{2}$ is the genetic variance of initial 150 individuals.

The genotypic values of the population improvement component significantly contributed to genetic improvement in the Inbred8 population. Additionally, we computed genetic gains in the population improvement component as follows:


(19) $$\begin{array}{c} G^{\mathrm{PIC}}(t)=\frac{u^{\mathrm{PIC}}(t)-\;u^{\mathrm{PIC}}(t=0)}{\sqrt{\sigma_{g}^{2}}} \end{array}$$

(20) $$\begin{array}{c} u^{\mathrm{PIC}}(t)=\underset{i\in [1,N]}{\max}\;u_{i}^{\mathrm{PIC}}(t) \end{array}$$

where $u_{i}^{\mathrm{PIC}}(t)$ represents the genotypic value of individual *i* in the population improvement component at cycle t(t=0,1,…,60), $u^{\mathrm{PIC}}(t=0)$ is equal to $u^{I8}(t=0)$ in Eq. 17, and *N* is the number of individuals in the population improvement component (N=150).

Moreover, the genetic variance in the population improvement component serves as a source of genetic improvement. The genetic variance of the population improvement components was computed as follows:


(21) $$\begin{array}{c} \sigma^{2}(t)=\frac{1}{N}\overset{N}{\underset{i=1}{\sum }}\;{\left(u_{i}^{\mathrm{PIC}}(t)-\bar{u^{\mathrm{PIC}}(t)}\right)}^{2} \end{array}$$

(22) $$\begin{array}{c} \bar{u^{\mathrm{PIC}}(t)}=\frac{1}{N}\overset{N}{\underset{i=1}{\sum }}\;u_{i}^{\mathrm{PIC}}(t) \end{array}$$

where $u_{i}^{\mathrm{PIC}}(t)$ remains the same as in Eq. 20.

## Results

### Genetic gain in the Inbred8 population

**Fig. 2.**
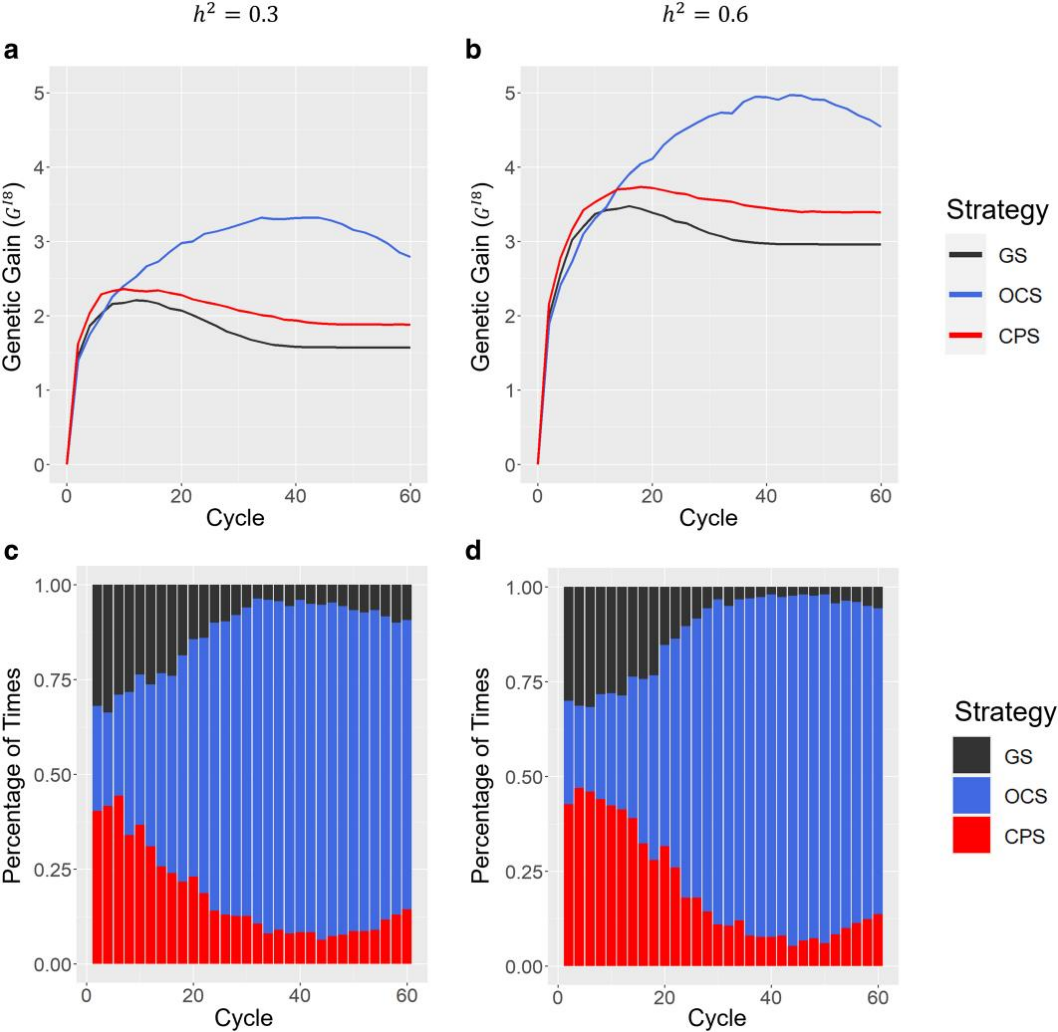
Comparison of 3 breeding strategies at the Inbred8 generation. GS, genomic selection; OCS, optimal cross selection, $t^{⋆}=60$, s=1, and $He^{⋆}=0.01He^{0}$; CPS, cross potential selection. a) Genetic gain ($G^{I8}$) in a scenario of $h^{2}=0.3$. b) Genetic gain ($G^{I8}$) in a scenario of $h^{2}=0.6$. c) The percentage of times that each breeding strategy showed the highest genetic improvement for each cycle in a scenario of $h^{2}=0.3$. d) The percentage of times that each breeding strategy showed the highest genetic improvement for each cycle in a scenario of $h^{2}=0.6$.

Figure 2a and b illustrates the genetic gains of the Inbred8 generation under each scenario $\left(h^{2}=0.3\;\mathrm{or}\;0.6\right)$. In $h^{2}=0.6$, CPS exhibited the highest genetic gains among the 3 strategies (GS, OCS, and CPS) in the early cycles (2≤t≤12) (Fig. 2b). In particular, in cycle t=8, CPS outperforms GS and OCS by 7 and 10%, respectively. In $h^{2}=0.3$, the advantage of the short-term improvement in the CPS was lower than that in the scenario of $h^{2}=0.6$ (Fig. 2a). However, CPS exhibited the highest genetic gain among the 3 strategies until t=8. The CPS percentages showed that the highest genetic gain at cycle t=6 among 300 independent breeding simulations were 0.44 and 0.46 in $h^{2}=0.3$ and $h^{2}=0.6$ (Fig. 2b and d). CPS continuously outperformed GS in each scenario, but OCS surpassed CPS regarding long-term genetic improvement (Fig. 2). The OCS reached $G^{I8}(t=44)=5.0$, which was 35% higher than the $G^{I8}(t=18)=3.7$ reached by the CPS in $h^{2}=0.6$ (Fig. 2b).

### Genetic gain and variance in population improvement component

**Fig. 3.**
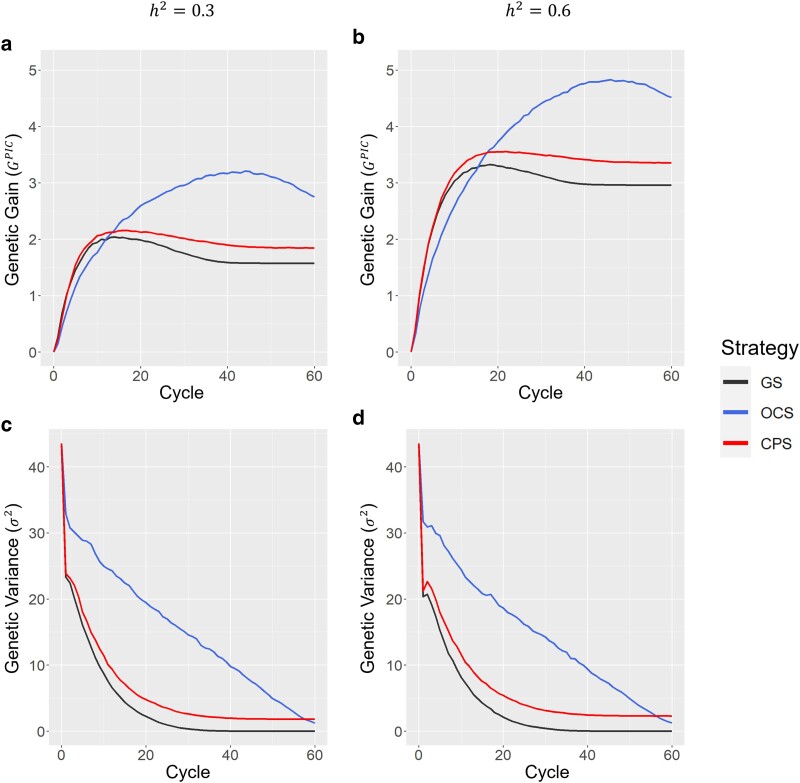
Comparison of 3 breeding strategies in the genetic improvement component. GS, genomic selection; OCS, optimal cross selection, $t^{⋆}=60,\;s=1$, and $He^{⋆}=0.01He^{0}$; CPS, cross potential selection. a) Genetic gain ($G^{\mathrm{PIC}}$) in a scenario of $h^{2}=0.3$. b) Genetic gain ($G^{\mathrm{PIC}}$) in a scenario of $h^{2}=0.6$. c) Genetic variance ($\sigma^{2}$) in a scenario of $h^{2}=0.3$. d) Genetic variance ($\sigma^{2}$) in a scenario of $h^{2}=0.6$.

Figure 3a and b shows the genetic gain in the population improvement component, which followed a trend similar to that of the Inbred8 generation (Fig. 2a and b). In $h^{2}=0.6$, CPS achieved 5 and 25% greater genetic gains than GS and OCS, respectively, at cycle t=9 (Fig. 3b). CPS maintained higher genetic variance than GS over all cycles, whereas OCS maintained higher genetic variance than CPS in each scenario (Fig. 3c and d). The linear trajectory of genetic variance in the OCS reflects the shape parameter (s=1) in Eq. 8. GS and OCS nearly exhausted all genetic variance in the final cycle, whereas CPS retained 5% of genetic variance from the initial cycle (t=0).

### Allele states

**Fig. 4.**
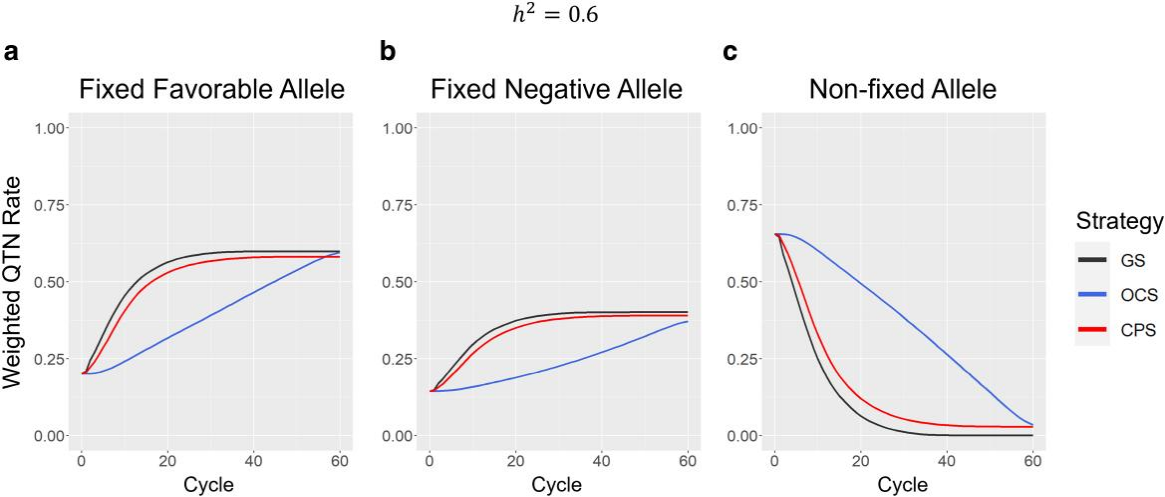
Allele states in the population improvement component for each strategy in a scenario of $h^{2}=0.6$. GS, genomic selection; OCS, optimal cross selection, $t^{⋆}=60$, s=1, and $He^{⋆}=0.01He^{0}$; CPS, cross potential selection. The weighted QTN rate of each category is calculated as the sum of the absolute effects of corresponding QTN for each category divided by the sum of the absolute effects of all QTN. a) Fixed favorable allele. b) Fixed negative allele. c) Non-fixed allele.

Fig. 4 shows the allele states of the population improvement component for each breeding strategy in $h^{2}=0.6$. Only the case in $h^{2}=0.6$ is shown because the results were almost identical for the cases in $h^{2}=0.3$ and $h^{2}=0.6$. The case in $h^{2}=0.3$ is shown in Supplementary Fig. 1. The weighted QTN rate of each category (fixed favorable, fixed negative, and non-fixed alleles) was computed as the sum of the absolute effects of the corresponding QTN for each category divided by the sum of the absolute effects of all QTNs. GS and CPS showed similar trends, but OCS did not fix as many favorable and negative alleles (Fig. 4a and b). OCS retained many alleles in non-fixed states to avoid losing any favorable alleles (Fig. 4c). In OCS, the alleles were gradually fixed for the target generation ($He^{⋆}=60$) in Eq. 8 (Fig. 4a and b).

## Discussion

### Short-term genetic improvement

In this study, we developed a novel breeding strategy called CPS, which focuses on selecting crossing pairs based on the expected value of a superior fraction of the progeny of the target generation. CPS demonstrated the greatest short-term genetic improvement among the 3 strategies (GS, OCS, and CPS) under both scenarios ($h^{2}=0.3\;\mathrm{and}\;0.6$) (Fig. 2). This finding aligns with the outcomes reported by des Déserts *et al*. (2023), in which crossing pairs selected using UC resulted in superior genetic improvements in the F_5_ population compared to those selected using GEBVs. Additionally, a breeding strategy combined UC and CPS (i.e. replacing Eq. 5 by Eq. 15) can balance the short-term and long-term genetic improvements (Allier, *et al*. 2019b), but adjusting parameters in OCS ($t^{⋆}$, *s*, and $He^{⋆}$) requires a long time. In CPS, because there is no parameter that requires adjusting, it can be quickly applied to breeding programs. In Allier *et al*. (2019b), UC was proven useful in recurrent selection, using DHs produced after mating as the next mating population. However, the combination of UC and rapid recurrent selection using high heterozygous individuals for rapid varietal development has not yet been explored. Considering the imperative to expedite the production of novel varieties through breeding, short-term genetic improvement is essential. Hence, we posit that CPS is a valuable breeding strategy capable of delivering significant short-term genetic improvements. Regarding short-term genetic improvements, the usefulness of CPS was high when the heritability of the target trait was higher than when the heritability of the target trait was lower (Fig. 2a and b). Previous research has indicated that the efficiency of strategies such as UC is contingent on the accuracy of progeny variance prediction (des Déserts *et al*. 2023). To maximize the usefulness of CPS in plant breeding programs, it is necessary to build a highly accurate GP model.

### Genetic variance

Genetic variance and diversity in breeding populations are fundamental sources of genetic improvement (Jannink *et al*. 2010; Sanchez *et al*. 2023, 2024). OCS effectively maintains genetic variance by integrating a penalty term based on the degree of kinship among selected individuals according to its selection criterion (Gorjanc *et al*. 2018; Lehermeier, *et al*. 2017; Allier *et al*. 2019b) In our study, OCS consistently maintained high genetic variance and ultimately achieved the most significant genetic gain among the 3 strategies by the final cycle (Figs. 2 and 3). In contrast, CPS, which did not explicitly consider genetic diversity, managed to sustain a higher genetic variance than GS (Fig. 3b). Recurrent selection utilizing UC resulted in a higher Bulmer effect (Bulmer 1971) than that using GEBVs, and this high Bulmer effect contributed to the high genetic variance in a breeding strategy utilizing UC (Allier, *et al*. 2019b). In the CPS, the selection of crossing pairs is predicated on the UC, derived from genetic variance in the Inbred8 generation. As the genetic distance between the 2 individuals selected as crossing pairs increased, the genetic variance of the Inbred8 population also increased. Consequently, the selection of crossing pairs with greater genetic distances likely contributed to the maintenance of high genetic variance (Fig. 3c and d). The higher heterozygosity observed in CPS than in GS further supports the tendency of CPS to favor crossing pairs with greater genetic distances (Fig. 4c). Due to its sustained genetic variance, CPS consistently outperformed GS even in later cycles (Fig. 2).

CPS retained some genetic variance even in the final cycle, regardless of the differences in heritability (Fig. 3c and d). The CPS cannot use genetic variance because of its inability to accurately assess the potential of certain crosses in later cycles. Even in the later cycles, the true genotypic values of the Inbred8 population followed a normal distribution. However, in later cycles, the GEBVs of the Inbred8 progeny produced from some crosses deviated from normal distribution because of the fixation of numerous alleles (Supplementary Fig. 2). Since the genetic variance of the progeny has a significant effect on UC values, CPS tends to select crosses that will maintain heterozygous allele sets rather than fix them. In such cases, the GEBV in the Inbred8 generation did not follow a normal distribution, which led to an overestimation of the potential of some crosses and their selection as high-potential candidates (Supplementary Fig. 3). Indeed, the UC of the 10 crosses selected in the final cycle were overestimated (Supplementary Fig. 4).

Although CPS may not be optimal for long-term breeding programs, we believe that this overestimation is not a critical issue. CPS is a strategy for short-term genetic improvement and it was shown to outperform GS regarding short-term genetic improvement in each scenario $\left(h^{2}=0.3\;\mathrm{or}\;0.6\right)$. Additionally, implementing a breeding scheme spanning over 10 years (20 cycles) to produce varieties requires significant time and cost. In actual soybean breeding, it takes approximately ≥5 years to evaluate productivity and stability at many sites. Consequently, we believe that CPS is well-suited for short-term genetic improvements, whereas OCS is better suited for long-term genetic improvements. CPS lost more useful alleles than OCS because it did not consider genetic diversity (Fig. 4). In the recurrent selection, since lost favorable alleles cannot be collected again, it is important to avoid losing useful alleles for long-term genetic improvement. As there is a tradeoff between short- and long-term genetic improvements, it is necessary to select an appropriate breeding strategy based on the resources provided to the breeding program, such as time and money. In this context, CPS is a suitable breeding strategy for the rapid production of varieties with limited time and money.

## Conclusion

In this study, we assessed the efficacy of CPS as a novel breeding strategy for rapid varietal development. A breeding strategy that allows to produce varieties as quickly as possible is required to respond immediately to changes in climate and social demands. Our findings highlight CPS as a valuable approach for achieving short-term genetic improvement, thereby facilitating the expedited production of novel varieties. The usefulness of the CPS was partly determined by the prediction accuracy of the target trait (Fig. 2a and b). To effectively implement CPS in real-world breeding programs, developing a highly accurate GP model is imperative.

## Supplementary Material

jkae224_Supplementary_Data

## Data Availability

All datasets and source codes for the breeding simulations are available from the repository in the GitHub, “https://github.com/Sakuraikengo/CPS.” Supplemental material available at G3 online.
