# Supplementary material for: Cross potential selection: a proposal for optimizing crossing combinations in recurrent selection using the usefulness criterion of future inbred lines
Source: G3 (Bethesda). 2024 Sep 23;14(11):jkae224. doi: 10.1093/g3journal/jkae224 (PMC11540310; doi:10.1093/g3journal/jkae224)
Supplement: jkae224_Supplementary_Data [file jkae224_supplementary_data.zip › Figure_S1_G3-2024-405208.docx]

**Figure S1**. Allele states in the population improvement component for each strategy in a scenario of $h^{2}=0.3$. GS: genomic selection, OCS: optimal cross selection, $t^{\star}=60$, $s=1$, and ${He}^{\star}=0.01{He}^{0}$,, and CPS: cross potential selection. The weighted QTN rate of each category is calculated as the sum of the absolute effects of corresponding QTN for each category divided by the sum of the absolute effects of all QTN. (A) Fixed favorable allele, (B) Fixed negative allele, (C) Non-fixed allele.
