# Supplementary material for: Cross potential selection: a proposal for optimizing crossing combinations in recurrent selection using the usefulness criterion of future inbred lines
Source: G3 (Bethesda). 2024 Sep 23;14(11):jkae224. doi: 10.1093/g3journal/jkae224 (PMC11540310; doi:10.1093/g3journal/jkae224)
Supplement: jkae224_Supplementary_Data [file jkae224_supplementary_data.zip › Figure_S2_G3-2024-405208.docx]

**
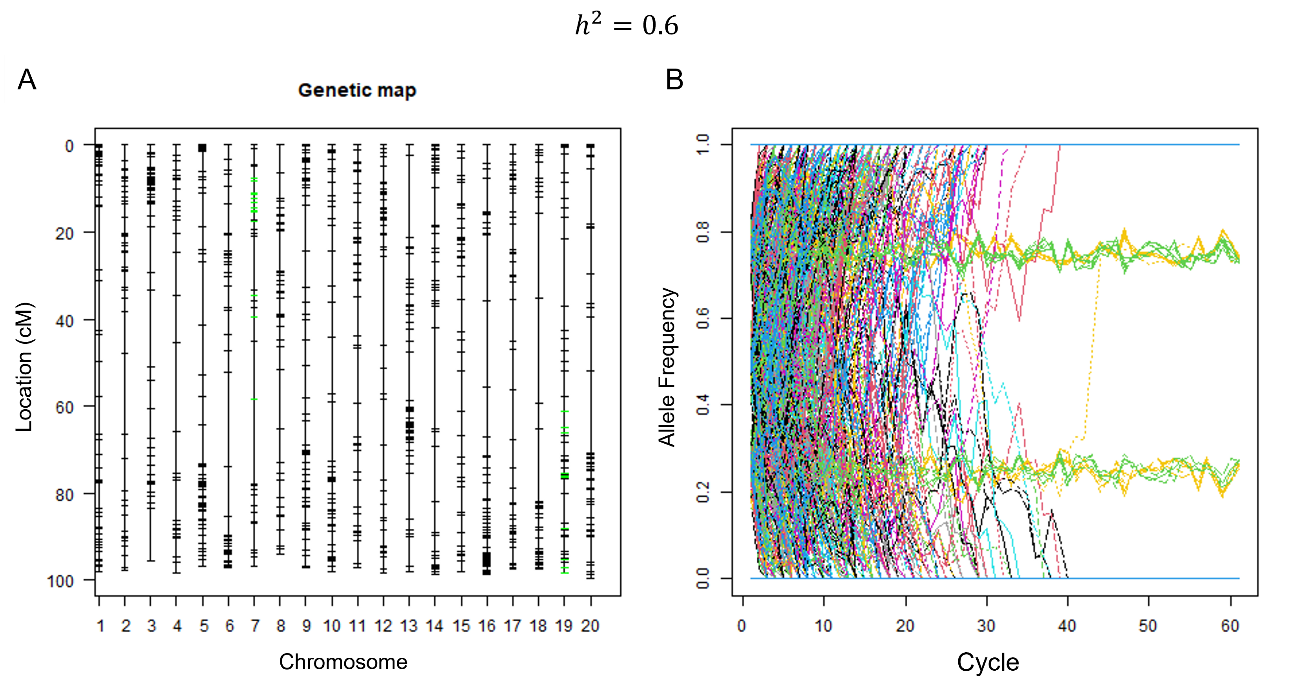
**

**Figure S2**. Allele states in the genetic improvement component of CPS (cross potential selection) at the final cycle ($t=60$) in a scenario of $h^{2}=0.6$. The result of 1^st^ breeding simulation among 300 independent breeding simulations. (A) 1,000 QTN locations in 20 chromosomes. Green line: not fixed QTN, black line: fixed QTN. (B) Time-series change of desirable 1,000 QTNs frequencies. Each line shows each QTN. 1 means QTN is fixed toward desirable direction and 0 means QTN is fixed toward undesirable direction. Each color shows each chromosome.
