# Supplementary material for: Cross potential selection: a proposal for optimizing crossing combinations in recurrent selection using the usefulness criterion of future inbred lines
Source: G3 (Bethesda). 2024 Sep 23;14(11):jkae224. doi: 10.1093/g3journal/jkae224 (PMC11540310; doi:10.1093/g3journal/jkae224)
Supplement: jkae224_Supplementary_Data [file jkae224_supplementary_data.zip › Figure_S3_G3-2024-405208.docx]

**
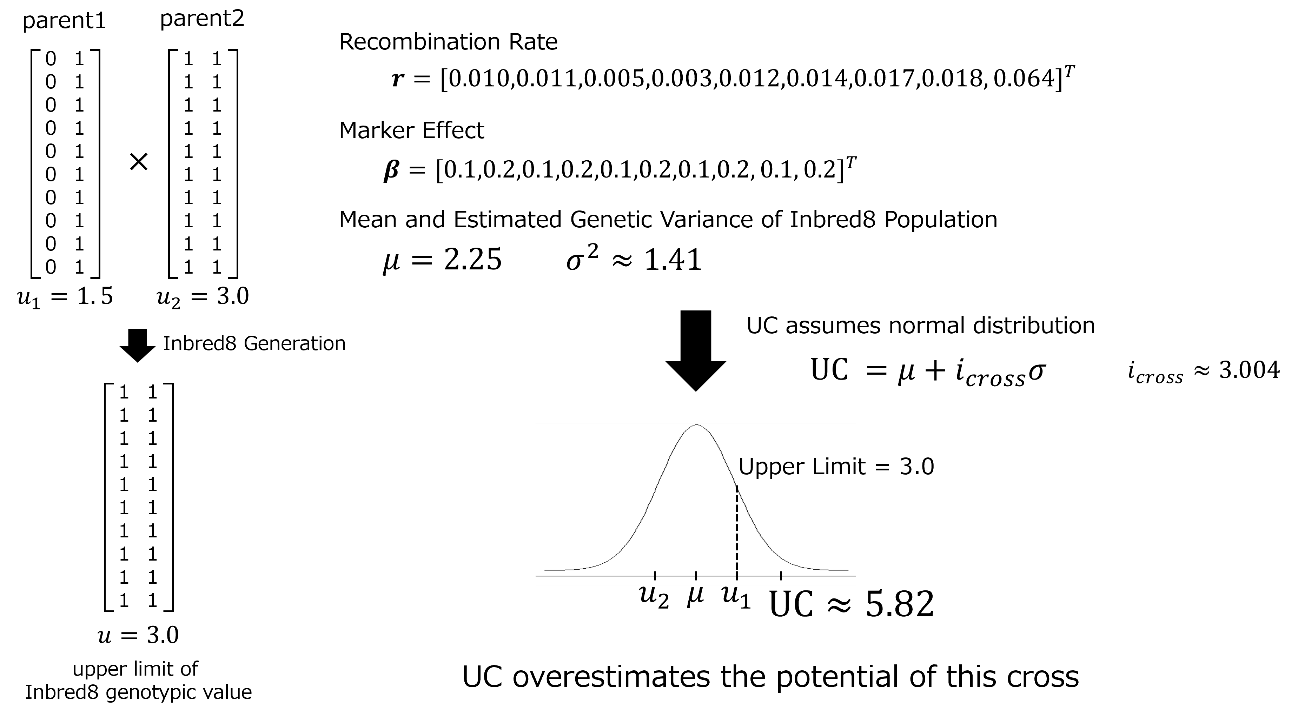
**

**Figure S3**. The mechanism of overestimation in CPS (cross potential selection). For simplicity, we consider only 10 QTNs. UC of this cross is close to 5.82, but the upper limit of Inbred8 genotypic value is 3.0. In this way, UC overestimates the potential of some crosses.
