# Supplementary material for: Cross potential selection: a proposal for optimizing crossing combinations in recurrent selection using the usefulness criterion of future inbred lines
Source: G3 (Bethesda). 2024 Sep 23;14(11):jkae224. doi: 10.1093/g3journal/jkae224 (PMC11540310; doi:10.1093/g3journal/jkae224)
Supplement: jkae224_Supplementary_Data [file jkae224_supplementary_data.zip › Figure_S4_G3-2024-405208.docx]

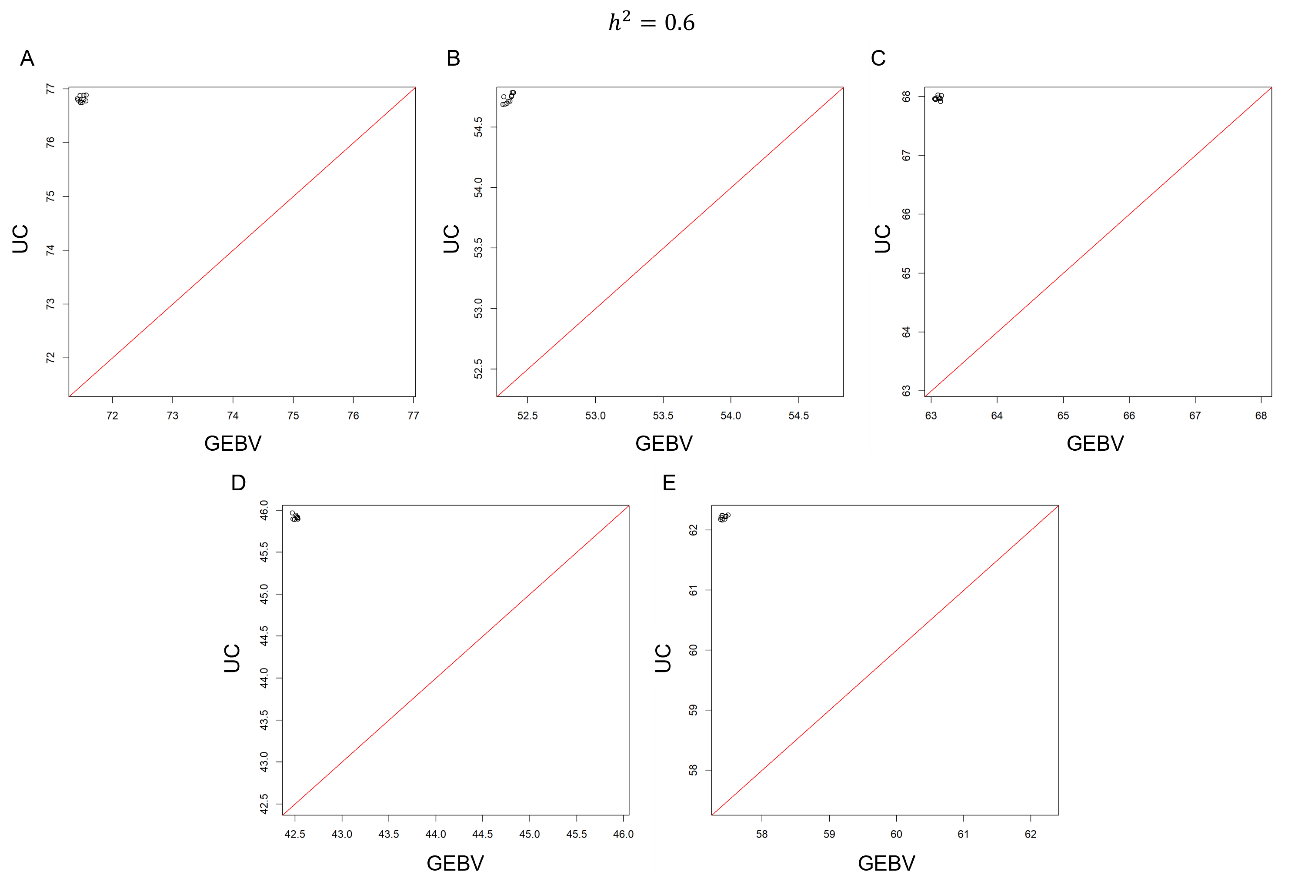


**Figure S4**. Relationship between UC calculated in Eq.13 and maximum genomic estimated breeding values (GEBVs) of Inbred8 individuals from selected 10 crosses respectively at the final cycle in a scenario of $h^{2}=0.6$. The results of 1^st^ to 5^th^ breeding simulations among 300 independent breeding simulations. Each plot indicates selected each cross and the number of crosses is 10. In each subfigure, all plots have the same value, but to visualize all plots a small amount of noise is added to all plots.
