# Supplementary material for: Cross potential selection: a proposal for optimizing crossing combinations in recurrent selection using the usefulness criterion of future inbred lines
Source: G3 (Bethesda). 2024 Sep 23;14(11):jkae224. doi: 10.1093/g3journal/jkae224 (PMC11540310; doi:10.1093/g3journal/jkae224)
Supplement: jkae224_Supplementary_Data [file jkae224_supplementary_data.zip › File_S1_G3-2024-405208.docx]

**Calculating the** $\boldsymbol{\Sigma}_{\boldsymbol{k}}$ **matrix for each cross**

We can consider the crosses between heterozygous individuals (not pure lines) as the 4-way crosses of pure lines. The way of calculating the $\boldsymbol{\Sigma}_{k}$ matrix was invented by Allier et al. (2019). We define that cross $k$ consisted of individual $a$ and $b$. Individual $a$ has $g_{1}$(gamet1) and $g_{2}$. Individual $b$ has $g_{3}$ and $g_{4}$. Following Allier et al. (2019), the ($j, l$) element of $\boldsymbol{\Sigma}_{k}$ can be computed as follows:

$$\begin{aligned} \Sigma_{jl}=\left( 1-2c_{jl}^{\left( 7 \right)}-\left( 0.5\left( 1-2c_{jl} \right) \right)^{7} \right)\Phi_{2jl})+\left( 1-c_{jl}^{\left( 7 \right)} \right)\left( 1-2c_{jl} \right)\Phi_{1jl}\#(S1) \end{aligned}$$

$$\begin{aligned} c_{jl}^{\left( 7 \right)}=\frac{2c_{jl}}{1+2c_{jl}}\left( 1-{0.5}^{7}\left( 1-2c_{jl} \right)^{7} \right)\#(S2) \end{aligned}$$

where $\Phi_{1 jl}=D_{jl}^{12}+D_{jl}^{34}$ is the sum of disequilibrium parameter between alleles $j$ and $l$ in pairs of gametes within each individual $a$ ($g_{1}$ and $g_{2}$) and $b$ ($g_{3}$ and $g_{4}$), $\Phi_{2 jl}=D_{jl}^{14}+D_{jl}^{13}+D_{jl}^{24}+D_{jl}^{23}$ is the sum of disequilibrium parameter between parental alleles $j$ and $l$ in pairs of gametes among individuals $a$ and $b$ ($g_{1}$ and $g_{4}$, $g_{1}$ and $g_{3}$, $g_{2}$ and $g_{4}$, and $g_{2}$ and $g_{3}$). $D_{jl}^{12}$ denotes the linkage disequilibrium between parental alleles $j$ and $l$ in the pair of $g_{1}$ and $g_{2}$ which can be computed as $D_{jl}^{12}=\frac{1}{16}{[(\boldsymbol{X}_{1}-\boldsymbol{X}_{2}){(\boldsymbol{X}_{1}-\boldsymbol{X}_{2})}^{T}]}_{jl}$. $\boldsymbol{X}_{s}$ is the $(L\times1)$ length allele state vector of gamet $s$, with the $j^{th}$ element coded as 1 or -1 for the alleles “A” or “a” at marker $j$. $c_{jl}$ is the recombination frequency between alleles $j$ and $l$ calculated from the distance of linkage map position $d_{jl}$ in Morgan as $c_{jl}=\frac{1}{2}(1-e^{-2d_{jl}})$ (Haldane, 1919). When $j$ and $l$ refer to parental alleles at the same marker, it holds $d_{jl}=c_{jl}=0$. This paragraph refers to material methods in Allier et al. (2019).

**Calculating the** $\sigma_{i}$ **for each individual**

Following the methodology outlined by Allier et al. (2019), the genetic variance of the Inbred8 population for each individual was computed using the SNP marker effect and score.

$$\begin{aligned} \sigma_{i}^{2}=\boldsymbol{\beta}^{T}\boldsymbol{\Sigma}_{i}\boldsymbol{\beta}\#\left( S3 \right) \end{aligned}$$

where $\boldsymbol{\beta}$ is the SNP marker effect vector $(L\times1)$, and $\boldsymbol{\Sigma}_{i}$ is the variance-covariance matrix $(L\times L)$, computed from the SNP marker score of individual $i$. Each element of $\boldsymbol{\Sigma}_{i}$ represents the variance or covariance between two markers at the Inbred8 generation. We can consider a selfing of each individual as a cross by itself. We can compute the $\boldsymbol{\Sigma}_{i}$ in the same way to compute the $\boldsymbol{\Sigma}_{k}$ in Eq.S1. Individual $a$ and $b$ of cross $k$ correspond to individual $i$. The ($j, l$) element of $\boldsymbol{\Sigma}_{i}$ can be computed as follows:

$$\begin{aligned} \Sigma_{jl}=\left( 1-2c_{jl}^{\left( 6 \right)}-\left( 0.5\left( 1-2c_{jl} \right) \right)^{6} \right)\Phi_{2jl})+\left( 1-c_{jl}^{\left( 6 \right)} \right)\left( 1-2c_{jl} \right)\Phi_{1jl}\#(S4) \end{aligned}$$

$$\begin{aligned} c_{jl}^{\left( 6 \right)}=\frac{2c_{jl}}{1+2c_{jl}}\left( 1-{0.5}^{6}\left( 1-2c_{jl} \right)^{6} \right)\#\left( S5 \right) \end{aligned}$$

where $\Phi_{1 jl}$, $\Phi_{2 jl}$, $c_{jl}$ are the same to the Eq.S1 and S2.

Reference

Allier, A., Moreau, L., Charcosset, A., Teyssèdre, S., & Lehermeier, C. (2019). Usefulness criterion and post-selection parental contributions in multi-parental crosses: Application to polygenic trait introgression. *G3: Genes, Genomes, Genetics*, *9*(5), 1469–1479. https://doi.org/10.1534/g3.119.400129

Haldane, J. B. S. (1919). The combination of linkage values and the calculation of distances between the loci of linked factors. *Journal of Genetics*, *8*, 299–309.
